# Serological and vaccine evaluation for hepatitis B among Community Health Workers

**DOI:** 10.1590/1518-8345.6107.3765

**Published:** 2023-01-30

**Authors:** Tauana de Souza Amaral, Clery Mariano da Silva Alves, Fabiana Ribeiro Rezende, Karlla Antonieta Amorim Caetano, Anaclara Ferreira Veiga Tipple

**Affiliations:** 1 Universidade Federal de Goiás, Goiânia, GO, Brazil.; 2 Scholarship holder at the Fundação de Amparo à Pesquisa no Estado de Goiás, Brazil.; 3 Universidade Federal de Goiás, Faculdade de Enfermagem, Goiânia, GO, Brazil.

**Keywords:** Community Health Workers, Hepatitis B, Hepatitis B Antibodies, Vaccination, Occupational Health, Occupational Exposure, Agentes Comunitários de Saúde, Hepatite B, Anticorpos Anti-Hepatite B, Vacinação, Saúde do Trabalhador, Exposição Ocupacional, Agentes Comunitarios de la Salud, Hepatitis B, Anticuerpos contra la Hepatitis B, Vacunación, Salud Laboral, Exposición Profesional

## Abstract

**Objective:**

to identify the vaccination and serological status against hepatitis B among community health workers; to vaccinate against hepatitis B virus and to evaluate the immune response of susceptible workers.

**Method:**

phase I, cross-sectional and descriptive study, among community health workers in a capital city of the Midwest region, through a self-administered questionnaire, checking of vaccination cards, and blood collection for testing of serological markers for hepatitis B. Phase II, cohort study carried out in vaccinated non-immune workers identified in phase I. They received one dose of vaccine (challenge dose) and serological testing.

**Results:**

a total of 109 workers participated in the study. Most had vaccination record (97; 89.0%) and vaccination completeness (75; 77.3%), while the isolated anti-HBs (Antibodies against hepatitis B virus) marker was detected in 78 (71.6%) workers. The prevalence of hepatitis B virus exposure was 8.2%. Of the ten non-immune vaccinated workers, after challenge dose, one remained susceptible.

**Conclusion:**

although most workers are vaccinated and show immunological response to hepatitis B, susceptibility after challenge dose was identified. Therefore, it is necessary to have a surveillance program of the vaccination situation and serological status for this virus, to promote these workers’ safety.

## Resumo

**Objetivo:**

identificar a situação vacinal e sorológica contra hepatite B entre agentes comunitários de saúde; vacinar contra o vírus da hepatite B e avaliar a resposta imunológica dos agentes susceptíveis.

**Método:**

fase I, estudo transversal e descritivo, entre agentes comunitários de saúde de uma capital da região Centro-oeste, por meio de questionário autoaplicável, conferência do cartão vacinal e coleta de sangue para testagem dos marcadores sorológicos para hepatite B. Fase II, estudo de coorte realizado em trabalhadores vacinados não imunes e identificados na fase I. Estes receberam uma dose da vacina (dose desafio) e teste sorológico.

**Resultados:**

participaram do estudo 109 agentes. A maioria tinha registro de vacinação (97; 89,0%) e completude vacinal (75; 77,3%), já o marcador anti-HBs (anticorpos contra o vírus da hepatite B) isolado foi detectado em 78 (71,6%) agentes. A prevalência de exposição ao vírus da hepatite B foi de 8,2%. Dos dez agentes vacinados não imunes, após a dose desafio, um permaneceu susceptível.

**Conclusão:**

apesar da maioria dos trabalhadores estarem vacinados e apresentarem resposta imunológica para hepatite B, a suscetibilidade após a dose desafio foi identificada. Portanto, é necessário que haja um programa de vigilância da situação vacinal e estado sorológico para este vírus, para promover a segurança destes trabalhadores.

## Resumen

**Objetivo:**

identificar la situación de la vacunación y serología contra la hepatitis B entre agentes comunitarios de la salud, vacunar contra el virus de la hepatitis B y evaluar la respuesta inmunológica de los agentes susceptibles.

**Método:**

fase I, estudio transversal y descriptivo, entre agentes comunitarios de la salud de una capital de la región centro oeste, por medio de cuestionario autoadministrado, verificación del carné de vacunación y extracción de sangre para comprobar los marcadores serológicos para la hepatitis B. Fase II, estudio de cohorte realizado en trabajadores vacunados no inmunes e identificados en la Fase I; estos recibieron una dosis de la vacuna (dosis de desafío) y realizaron el test serológico.

**Resultados:**

participaron del estudio 109 agentes. La mayoría tenía registro de vacunación (97; 89,0%) y de cobertura de vacunación (75; 77,3%); el marcador anti-HBs (Anticuerpos contra el virus de la hepatitis B) aislado fue detectado en 78 (71,6%) de los agentes. La prevalencia de exposición al virus de la hepatitis B fue de 8,2%. De los diez agentes vacunados no inmunes, después de la dosis desafío, uno permaneció susceptible.

**Conclusión:**

a pesar de que la mayoría de los trabajadores estaban vacunados y presentaron respuesta inmunológica para la hepatitis B, la susceptibilidad, después de la dosis desafío, fue identificada. Por tanto, es necesario que exista un programa de vigilancia de la situación de vacunación y estado serológico para este virus, para promover la seguridad de estos trabajadores.

**Highlights**

(1) Most Community Health Workers (CHWs) had vaccination completeness (77.3%).

(2) Isolated Anti-HBs was detected in 71.6% of CHWs.

(3) The prevalence of hepatitis B virus exposure was 8.2%.

(4) A non-immune CHW remained susceptible after challenge dose.

(5) Surveillance program is necessary for occupational safety of CHWs.

## Introduction

Community Health Workers (CHWs) make up the multi-professional health team in many countries around the world^1^. The International Labor Organization defines CHWs as professionals who provide care to the population through “referral and follow-up, case management, basic preventive health services, and home visiting services to specific communities”^2^. 

In performing their work activities, these workers are exposed to risks, including exposure to biological material^3^, and the hepatitis B virus (HBV) is of epidemiological importance for this group^4^. In 2019, approximately 296 million people were chronic carriers of the HBV^5^ and in some groups, such as health care workers (HCWs), the prevalence was higher^6^.

It is known that vaccination is the main preventive measure against HBV, therefore, the Brazilian National Immunization Program, since 1993, has made the vaccine available for free for all HCWs with a schedule of three doses at intervals of zero, one and six months^7^. Healthcare professionals present different prevalence of hepatitis B immunization in the country^8^ ^-^ ^11^. 

For HCWs, in addition to vaccination, international and national organizations recommend a test to confirm the vaccine-induced immune response, the antibodies to hepatitis B virus surface antigen (anti-HBs), 30 to 60 days after completion of the vaccination schedule^12^ ^-^ ^16^. Antibody detection ≥ 10 milli-international units (mUI)/mL ensures lifelong worker protection^17^; however, studies show low adherence to this test by Brazilian HCWs^11^ ^,^ ^18^.

The cost of implementing testing for anti-HBs after completion of the regimen is low compared to post-exposure prophylactic management of HBV^19^. In addition, health care facilities that jointly adopt anti-HBs testing in surveillance programs and the vaccine have higher HCW vaccination coverage than facilities that do not offer the test^20^. And this ensures greater safety for the execution of the labor activities of these workers. 

Regarding CHWs, there is still a gap in knowledge about HBV-related health conditions among these workers, who have been working in primary care since 1991. Worldwide, there are also few studies conducted with this professional category. Thus, knowing the vaccination and serological status of CHWs against HBV allows the development of protection strategies for these workers exposed to this virus. 

The objectives of the study were: to identify the vaccination and serological status against hepatitis B among Community Health Workers; to vaccinate against hepatitis B virus and to evaluate the immune response of susceptible workers.

## Method

### Study design and site

This is an epidemiological study, guided by the STROBE (Strengthening the Reporting of Observational Studies in Epidemiology) initiative, with two methodological designs. Phase I consisted of a cross-sectional and descriptive study, carried out with CHWs with work assignment in a large capital city in the Midwest region of Brazil. Phase II was a cohort consisting of CHWs included in phase I and eligible to receive the challenge dose of hepatitis B vaccine. 

Goiânia, capital of Goiás, is a city with approximately 1,536,097 inhabitants, located in the central region of Brazil^21^. Primary Health Care was established in the country in 1994, and is carried out in the the Family Health Centers (FHC)^22^. Currently, Goiânia has 62 FHC, divided into seven Health Districts with 896 CHWs^23^.

This study was conducted in the Western Health District whose region, according to the Primary Care Coordination of the Municipal Health Secretariat, is the largest in terms of coverage area and number of FHC (14 in total). Moreover, the number of 172 CHWs in the district is consistent with the number of teams and population of the territory covered.

### Study period and population

The cross-sectional study was conducted from July to December 2019 and the cohort from January to May 2020. In phase I, CHWs aged 18 years or older were included and those who were on vacation and/or leave on the scheduled dates were excluded. Therefore, of the 172 CHWs, 36 were on vacation or leave and were excluded from the study. The inclusion criteria for the cohort (phase II) were: CHWs who had three doses of the hepatitis B vaccine and anti-HBs titers <10 mIU/mL in the serological test. Individuals in this situation were eligible to receive the challenge dose^12^.

### Data collection

Initially, a virtual invitation was made by the managers of each unit to the active CHWs. Those who showed up at the scheduled date and place and agreed to participate in the study, after signing the Free and Informed Consent Term (FICT), were invited to answer the questionnaire. This questionnarie contained questions about socio-demographic characteristics and knowledge about hepatitis B vaccination. After this step, the vaccination card was requested to check the hepatitis B vaccination record; in its absence, information was sought in the National Immunization Program Information System (NIPIS).

Finally, 8 milliliters (mL) of blood sample were collected through venipuncture in the upper limb for hepatitis B serology. All samples were tested for the following serological markers: hepatitis B surface antigen (HBsAg), antibodies to hepatitis B virus core protein (anti-HBc) and anti-HBs. Rapid Test from Bioclin, Brazil, was used for detecting HBsAg marker. The anti-HBc and anti-HBs markers were tested using the chemiluminescence method (Architect i1000TM, Abbott Diagnostics).

The test results were personally delivered to each CHW. Those who were not vaccinated or had an incomplete schedule were oriented to take a new three-dose schedule or to complete the schedule^12^ ^,^ ^16^. 

Subjects eligible for phase II received one dose of hepatitis B vaccine (“challenge dose”), conceptualized as administration of one dose of hepatitis B vaccine to screen for the presence of memory antibodies against possible exposure to the virus^24^. Thus, within 30 to 60 days after vaccination, a new blood sample was collected for serological testing for the anti-HBs marker. The test was performed using the electrochemiluminescence method (Cobas e 411TM, Elecsys Anti-HBs kit, Roche). Individuals who did not present protective anti-HBs titers were referred to the public health care network and oriented to continue hepatitis B vaccination.

### Study variables

The variables investigated in this study were: sex; age group; level of education; length of service as a CHW; complete hepatitis B vaccination schedule; report of anti-HBs testing; anti-HBs value; presentation of the hepatitis B vaccination card; time elapsed between the last dose of hepatitis B vaccine and the date of anti-HBs testing, and the serological response to the challenge dose (second anti-Hbs). 

Global prevalence for hepatitis B was considered to be positivity for the HBsAg and/or anti-HBc marker; immunized individuals, in turn, were defined by the presence of isolated anti-HBs with antibody titers ≥ 10 mUI/mL^24^.

### Data treatment and analysis 

Statistical Package for Social Science (SPSS) software version 21.0 was used for data analysis. Prevalence was estimated with 95% confidence intervals (CI). Positive predictive value, negative predictive value and specificity of the hepatitis B vaccination record were calculated. 

### Ethical aspects

This study was approved in Research Ethics Committee of the Clinical Hospital of the Federal University of Goiás, Certificate of Ethics Appreciation Presentation (CEAP): 41413015.6.0000.5078, opinion number 980.293 of 2015. For the extension of the objectives, an addendum was requested to the respective committee, approved under CEAP 41413015.6.0000.5078, opinion number 3,632,014 of 2019. 

## Results

Of the 172 CHWs belonging to the Western Health District, 109 (63.3%) participated in the study, 36 were on vacation or leave, 20 did not show up at the units on the collection days and seven refused. Regarding the socio-demographic characteristics, most CHWs were female (91.7%), aged 31 to 40 years (45.9%), had completed high school (44.0%) and had been working for less than five years (37.6%). When asked about the vaccination schedule against hepatitis B, 58.7% (64/109) CHWs said they had received the three doses of the vaccine; however, 48.4% (31/64) reported having been tested for anti-HBs test, 36.0% (23/64) did not perform it and 15.6% (10/64) did not inform. Of the CHWs who reported having been tested for anti-HBs, 74.2% (23/31) reported values ≥ 10 mIU/mL. 

The overall prevalence of hepatitis B among the investigated CHWs was 8.2%. In 78 (71.6%) CHWs, titers ≥ 10 mIU/Ml for anti-HBs were observed, in isolation, indicating previous hepatitis B vaccination. More than 20% were in susceptible condition to HBV with age ranging from 30 to 60 years.

It was possible to access information on hepatitis B vaccination records for 97 participants (89%). Of these, 77.3% (75/97) had a complete hepatitis B schedule, but only 35 of these CHWs reported having been tested after completion of the vaccination schedule. The positive predictive values and specificity of the hepatitis B vaccination record compared with the serological marker for hepatitis B were 74.7% and 38.7%, respectively (Table 1).

**Table 1:** Positives (PPV^*^) and Negatives Predictive Values (NPV^†^) of hepatitis B vaccine record compared with serological marker for hepatitis B, “isolated anti-HBs^‡^”, in 109 Community Health Workers in the municipality of Goiânia-GO. Goiânia, GO, Brazil, 2019-2020

Vaccination card	Isolated Anti-HBs^‡^		
	Positive	Negative	Total
Complete schedule	56^§^	19	75
Incomplete schedule or no card	22	12^ǁ^	34
Total	78^§^	31^ǁ^	109

*PPV 56/75 = 74.7%; ^†^NPV 12/34 = 35.3%; ^‡^anti-HBs = Antibodies against hepatitis B virus surface antigen; ^§^Sensitivity 56/78 = 71.8%; ^ǁ^Specificity 12/31 = 38.7%

**Figure 1:**
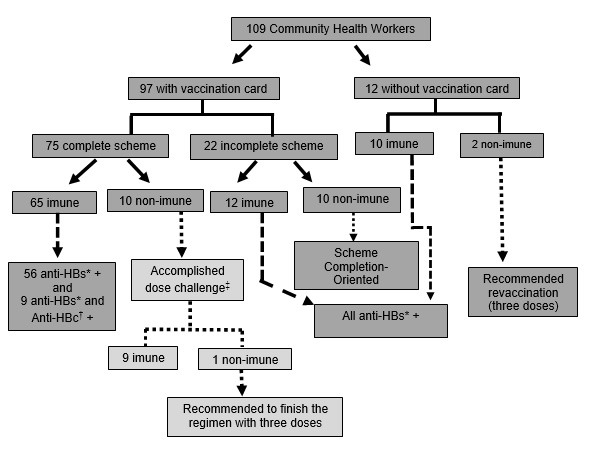
Flowchart of the presentation of the vaccination record and the association with serological markers of Community Health Workers (N= 109) of a health district of Goiânia. Goiânia, GO, Brazil, 2019-2020

After hepatitis B vaccination records were evaluated among those who received three doses of the vaccine, ten individuals who did not have protective titers against hepatitis B were identified and made up the cohort. One dose of vaccine was administered to all ten participants. The time between the last dose of hepatitis B vaccine received and the date of confirmatory anti-HBs testing ranged from six to 18 years. One individual only did not develop anti-HBs titers ≥ 10 mIU/mL (Figure 1).

* anti-HBs = Antibodies to the surface antigen of hepatitis B virus; ^†^anti-HBc = Antibodies to the core protein of hepatitis B virus; ^‡^According to Schillie, et al^12^

## Discussion

Since the introduction of the hepatitis B vaccine in Brazil in 1998, the epidemiological profile of HBV has been undergoing changes. Currently, a low prevalence for hepatitis B is found among the general population of the country^25^. On the other hand, some groups, such as CHWs, remain at risk for infection, considering their exposure characteristics and low vaccination rates. 

In this study, an HBV exposure rate of 8.2% (95% CI= 4.4%-15.5%) was found and the age of exposed individuals ranged from 31 to 61 years. Research conducted on 644 Spanish HCWs identified a prevalence for anti-HBc of 4.2% and with a progressive increase among older^26^. It is not possible to affirm that the exposure to the virus has happened during the work practice, but it is noteworthy that, during their activities, the CHWs may have been contaminated, as biological accidents are reported among this professional category^27^. Thus, there is a need for public policies for biological risk management in this group. Moreover, it is considered that these professionals should be prepared to face this risk, since their training, with the inclusion of biosafety^28^. It is noteworthy that most CHWs participating in this study had or were attending high school (technical or not) or higher education in courses related to the humanities sciences (20.2%), which confirms the lack of training on biological risk and safety measures.

Isolated anti-HBs titers were identified in 71.6% (n=78/109; 95% CI= 62.5%-79.2%) of participants, indicating previous hepatitis B vaccination. No studies were found that evaluated hepatitis B vaccination among CHWs by analysis of serological markers. However, a high percentage of protective titers against HBV are found among HCWs, as depicted in a study conducted in Ghana among 711 hospital workers, in which anti-HBs isolate was detected in 91.8% of the participating individuals^29^. 

It is known that proof of three doses of hepatitis B vaccine on the vaccination card is considered the gold standard for conferring vaccination status^30^. Ninety-seven CHWs (89.0%) had vaccine cards in this study and, of these, 75 had complete records of the three doses against hepatitis B, representing 68.8% (n=75/109; 95%CI= 59.6%-76.7%). The vaccination coverage, according to the record in the vaccination card, was similar to the serological testing performed, indicating a sensitivity of 71.8%. Despite the significant percentage of hepatitis B vaccination coverage, it was expected that all were vaccinated, since the vaccine has been implemented in Brazil for healthcare professionals since 1993^7^. Moreover, more than 90% of the participants joined the service less than 20 years ago.

No studies investigating vaccination coverage among CHWs through vaccination cards were identified in the literature consulted. Among the studies that showed vaccination rates reported by CHWs working in Primary Health Care through questionnaires, the percentages of vaccinated CHWs were below the value found in this study, 41.0%^31^, 65,4%^32^; however, in another study^8^, a slightly higher rate was identified, in which 84.1% of the CHWs reported immunization.

Therefore, among the individuals for whom the vaccination record was identified in the study (n=97), the fact that 22.7% (n=22/97) were developing their work activities at risk for hepatitis B draws attention, since incomplete doses significantly decrease the chances of the presence of protective titles against hepatitis B^29^.

This information is interesting, since one of the requirements for entry of any worker working in the health services is the presentation of a hepatitis B vaccination record^33^, and this may suggest a failure in the admission process and/or maintenance of the worker’s health. It is understood that it is necessary to establish a monitoring policy regarding the completion of the vaccination schedule by the CHWs for the protection of these workers. 

Based on serology, regardless of vaccination record, 22 individuals (20.1% n=22/109) were considered susceptible to HBV, a figure considered worrisome, since this group may have been exposed to body fluids in their work activities with risk of exposure to hepatitis B virus^34^. In an investigation on the profile of accidents with exposure to biological material that occurred in the southern region of the state of Minas Gerais, it was found that five CHWs (1.1%) had suffered accidents, among whom two had not been vaccinated against hepatitis B^27^. The chance of susceptible individuals contracting the virus ranges from 6%-30.0%^35^, thus, the possibility of seroconversion is a reality present in the CHWs’ work activities and may go unnoticed.

The age of the susceptible individuals ranged from 31 to 60 years. It can seen that some of these CHWs were born before the vaccine was implemented in the country^7^. However, the availability of the vaccine for free for over 20 years in the Unified Health System (UHS) for HCWs reveals the missed opportunities for vaccination among these workers, especially since they work in FHCs that have vaccine rooms as one of the essential environments in the unit’s structure^36^. 

Ten participants showed a vaccination card with a record of three doses of vaccine administration, but did not show antibody titers that could prove the effectiveness of the received schedule. However, after a challenge dose of hepatitis B vaccine, only one individual did not develop protective titers against hepatitis B, indicating good anamnestic response of the vaccine in these workers. 

International and national recommendations call for anti-HBs testing ideally 30 to 60 days after the completion of the vaccination schedule^12^ ^,^ ^16^. In this study, of the 75 who had a record of previous hepatitis B vaccination, 37.3% reported not having had an anti-HBs test after receiving the full vaccination schedule. International data show significant differences in hepatitis B vaccine adherence and effectiveness when professionals are followed continuously^20^. Thus, a labor monitoring policy may contribute to the prevention and control of hepatitis B.

In this study, hepatitis B vaccine non-responder status was not assessed. Two complete vaccination schedules followed by anti-HBs titers less than or equal to 10 mIU/mL within 30 to 60 days after completion of vaccination are required to confer vaccine non-responder status^12^. It is known that intrinsic, extrinsic, perinatal, environmental, behavioral, nutritional, vaccine and administration factors^37^ can affect the immunogenicity of an individual.

The findings of this study contribute to the advancement of scientific and health knowledge by highlighting and indicating the need for a surveillance program to monitor CHWs regarding their vaccination and serological status for hepatitis B, in order to ensure the safety of these workers. In this sense, they also contribute to the knowledge and practice of nursing, since they indicate the actions expected of the nurse team leader in the control of infectious diseases in Primary Health Care and promotion of occupational health.

This study has some limitations. The first is related to coverage, since the research, although conducted in a large region of the city of Goiânia, which includes 22.0% of all CHWs, was not performed in all FHCs in the city. The second limitation is related to vaccination coverage. Since it was not possible to obtain a photocopy of the vaccination card of all study participants, for those who did not present the card, we searched the National Immunization Program Information System in order to obtain information about the vaccination schedule of each worker. 

## Conclusion

In the evaluation of CHWs in work activities, it was identified that, of the 109 participants, 97 (89.0%) had physical records or NIPIS record, and 75 (77.3%) workers had complete schedule against hepatitis B virus. For those with a complete schedule, only 56 (74.7%) had isolated anti-HBs. Regarding the 10 with a full schedule but with absence of antibody titers, they received a dose of the vaccine, in which nine showed an anamnestic response and one, remained, non-immune. 

It is considered necessary to implement surveillance and monitoring program of the serological status of CHWs for HBV, which should include not only vaccination, but also anti-HBs testing after a complete schedule, since the biological risk is known. However, the adoption and description of measures in the policies of these workers, in Brazil, have not been observed yet.
